# Wound healing by topical application of *Momordica charantia* L. formulations on mice

**DOI:** 10.14202/vetworld.2021.2699-2704

**Published:** 2021-10-22

**Authors:** William Antonio Sagástegui-Guarniz, Carmen R. Silva-Correa, Víctor E. Villarreal-La Torre, María V. González-Blas, Walter O. Sagástegui-Guarniz, Abhel A. Calderón-Peña, Cinthya L. Aspajo-Villalaz, José L. Cruzado-Razco, Julio Hilario-Vargas

**Affiliations:** 1 Department of Pharmacology, School of Pharmacy and Biochemistry, National University of Trujillo, Trujillo, Peru; 2 Department of Biochemistry, School of Pharmacy and Biochemistry, National University of Trujillo, Trujillo, Peru; 3 Graduate School of Pharmacy and Biochemistry, National University of Trujillo, Trujillo, Peru; 4 Department of Biological Chemistry and Animal Physiology, School of Biological Science, National University of Trujillo, Trujillo, Peru; 5 Department of Physiology, School of Medicine, National University of Trujillo, Trujillo, Peru.

**Keywords:** histology, *Momordica charantia*, skin, topical administration, wound healing

## Abstract

**Background and Aim:**

*Momordica charantia* is mainly characterized by its antimicrobial and antioxidant properties. The current study aimed to evaluate the healing activity of gel and cream formulations based on *M. charantia* on induced wounds in mice.

**Materials and Methods:**

Acetonic extract of *M. charantia* was prepared and incorporated into gel and cream formulations. *Mus musculus* Balb/c (n=30) with induced injury were distributed into five groups: Group I (control – day 7), Group II (control – day 14), Group III (1% gel – day 7), and Group IV (1% gel – day 14) to which 1% *M. charantia* gel was dermally applied daily for 7 and 14 days, respectively, Group V (1% cream – day 7) and Group VI (1% cream – day 14) to which of *M. charantia* 1% cream were dermally applied daily for 7 and 14 days, respectively. Time of wound closure was determined during the experimentation; rats were euthanized with sodium pentobarbital 60 mg/kg/pc v.ip. for obtaining skin samples for histopathological analysis.

**Results:**

Groups IV and VI showed a higher percentage of wound closure on day 14, and in histopathological analysis, effect was greater in Group VI with the presence of fibroblasts and abundant collagen and elastic fibers.

**Conclusion:**

*M. charantia* gel and cream showed wound healing activity on induced wounded mice; the most effective treatment was *M. charantia* 1% cream formulation.

## Introduction

Wound healing is a complex and dynamic process that involves both local and systemic cellular and biochemical responses, which is supported by many cellular events that must be closely coordinated to efficiently repair damaged tissue [1,2]. This process begins with a fibroblastic stage where the wound area contracts [3]. The healing process is made up of different phases, such as hemostasis, inflammation, proliferation, and remodeling, in which the repair process requires the coordination of different cells, growth factors, and cytokines [4,5]. Skin wounds usually occur by accidental trauma such as burns, lacerations, or abrasions; which lead to complications in healing and infections [6]. Thus, adequate wound healing is essential for the restoration of interrupted anatomical continuity and altered functional status of the skin; but at the same time, it is sought that the treatment produces a rapid closure of the wound and a functional and esthetically satisfactory scar [7]. Wound healing depends on factors such as tissue repair ability, type and extent of damage, and overall health of the tissue [8].

However, wound healing medications remain unsatisfactory due to its high cost, low availability, limited efficacy, and various side effects [9]. Medicinal plants are widely used in folk medicine and many researchers have reported improvement in wound healing process by various plant extracts and isolated compounds; which, has provided healing wounds products more affordable with greater safety against hypersensitive reactions compared to synthetic pharmaceutical agents [10,11]. *Momordica charantia* L., belongs to the *Cucurbitaceae* family and is commonly known as bitter melon [12], karela, balsam pear, and bitter gourd. *M. charanti*a is a popular plant used for the treatment of conditions related to diabetes in indigenous populations from Asia, South America, India, the Caribbean, and East Africa [13,14].

*M. charantia* contains compounds such as momorcharin, momordenol, momordicillin, momordicinin I, II and III, momordine, momordolol, charantin, charine, cryptoxanthin, cucurbitans, cycloartenol, diosgenin, eleostearic acid, erythrodiol, galacturonic acids, gentisic acid, goyasaponin, and multiflorenol [15-18]; which are responsible for its various biological and pharmacological effects such as antidiabetic [19], antiulcer [20], antimicrobial [21], and antioxidant [22]. In addition, MAP30 protein that shows anti-HIV activity has also been identified in *M. charantia* [23].

Considering components of *M. charantia* L., this study was carried out to evaluate the effect of two formulations, one in gel and the other in cream based on *M. charantia*, on skin lesions induced in *Mus musculus* Balb/c to test its healing effect and set the scientific bases for subsequent research and applications in the health field.

## Materials and Methods

### Ethical approval

The study was approved by the Ethics Committee of the School of Pharmacy and Biochemistry of Universidad Nacional de Trujillo with the document COD. N°: P-007-19/C.FAC.FARM.

### Study period and location

The study was conducted from January to July 2020. All processes were performed in Toxicology Laboratory, School of Pharmacy and Biochemistry, National University of Trujillo.

### Biological material

*M. musculus* Balb/c (30-35 g) male, 14-16 weeks old, was used for this research. All mice were kept in individual cages under standard environmental conditions with 12:12 h dark photoperiod: light cycle and temperature of 22±2°C. They were given a balanced diet and water administered *ad libitum*.

### Vegetal material

Leaves of *M. charantia* L. were collected from Menocucho town, Trujillo Province, La Libertad Region, Peru. Taxonomic identification was carried out in the *Herbarium truxillense* at the Universidad Nacional de Trujillo, with code N° 59572.

### Preparation of the extract

Acetonic extract of *M. charantia* leaves was prepared, using 100 g of dried and pulverized leaves, leaving it to macerate in 1 L of acetone for 72 h with magnetic stirring. Then, it was brought to concentration in a rotary evaporator and in an oven until the dry extract was obtained, which was stored in amber containers and kept refrigerated at −10°C until use.

### Gel and cream formulations

The gel formulation was prepared based on Carbopol, triethanolamine, liquid petrolatum, propylene glycol, ethyl alcohol, and water. For cream formulation, the following inputs: Lane wax, liquid Vaseline, stearic acid, and cetyl alcohol were mixed over low heat, stirring until wax was completely melted, then the mixture of methylparaben, propylparaben, and glycerin was added until obtaining the base cream. Dry extract of *M. charantia* was added to both formulations until obtaining concentrations of 1%. Then, they were packaged, labeled, and stored at 25°C until use.

### Evaluation of healing activity

Before performing induction of wounds, all back of mice were depilated, 48 h later, lidocaine 2% cream was applied topically as an anesthetic, and a cut of 1 mm deep and 1 cm long, was made parallel to the longitudinal axis in the dorsal zone of each mouse. Each cut was measured with the help of a Vernier caliper [24].

Thirty *M. musculus* Balb/c were randomly distributed into six experimental groups with five specimens per group: Group I (control – day 7) and Group II (control – day 14) that did not receive any treatment, Group III (1% gel – day 7) and Group IV (1% gel – day 14) to which 1% gel of *M. charantia* was applied daily by topical route for 7 and 14 days, respectively; Group V (1% cream – day 7) and Group VI (1% cream – day 14) to which 1% cream of *M. charantia* was applied daily for 7 and 14 days, respectively. The wound healing process was recorded through the wound closure measurement parameter, evaluating during the 7 and 14 days of treatment.

The wound healing was measurement with a metric Vernier caliper and the percentage of wound closure was obtained with the formula:


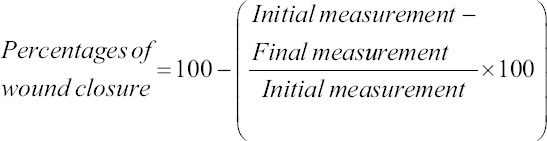


### Histopathological study

After 7 and 14 days of treatment, the experimental animals were euthanized using sodium pentobarbital 60 mg/kg intraperitoneal injection; then, skin samples were obtained by making cuts of 1 cm long and 0.5 cm long wide around the scar. Samples were preserved in sterile flasks with 10% formaldehyde solution for 8 days, and then, 3-5 μm parts were selected and fixed in paraffin [24]. Samples were stained with hematoxylin-eosin for reading under the microscope at ×400. The histopathology results were measured as differentiation, reepithelialization, and healing and graded as mild (+), moderate (++), and severe (+++).

### Statistical analysis

Graphs were prepared using Microsoft Office Excel^®^ (Microsoft Corp., USA) and data were subjected to an analysis of variance followed by *post hoc* Tukey test. Values were considered statistically significant at p<0.05.

## Results

### Wound healing evaluation

**Figure-1:**
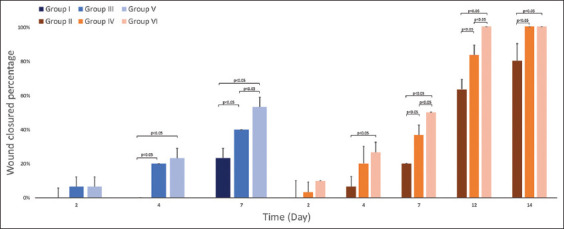
Evolution of percentage of wound closure in experimental groups. p<0.05, n=5.

The percentage of wound closure was used as a parameter evaluated to determine the evolution of the healing process. Percentages of wound closure on day 14 for Groups V (1% gel) and VI (1% cream) showed a significant difference compared to Group II (Control) (p<0.05) from day 4 after treatment administration, a similar effect was observed in groups of evolution of day 7 (Groups III and IV). In all groups, the greatest effect was observed in those who received cream formulation of *M. charantia* extract (Figure-1).

### Histopathological changes

**Figure-2:**
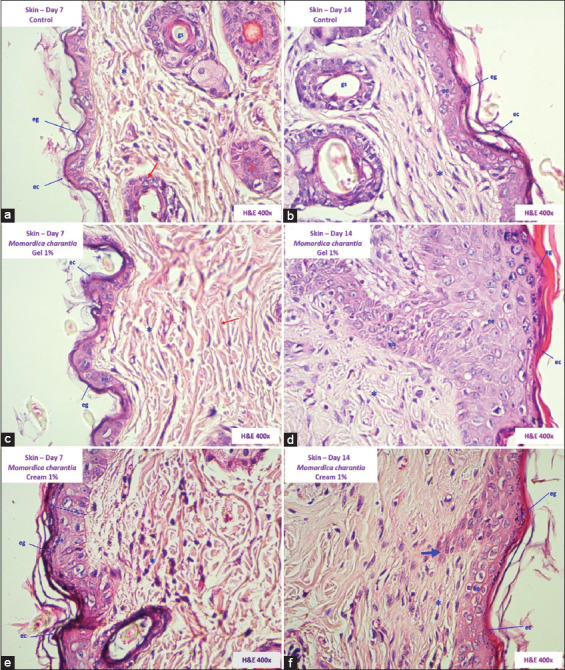
Histopathological sections of the skin of *Mus musculus* Balb/c. (a) Group I (control - day 7): differentiation (+). (b) Group II (control - day 14): re-epithelialization (++). (c) Group III (1% gel - day 7): Reepithelialization (++). (d) Group IV (1% gel - day 14): Healing (+++). (e) Group V (1% cream - day 14): Healing (+++). (f) Group VI (1% cream - day 14): Healing (+++). Keratinous stratum corneum (ec), stratum granulosum (eg), Langerhans cells in stratum spinosum (ee), fibroblasts (*), sebaceous glands (gs), papillary projections (arrow). (Stained with hematoxylin and eosin, × 400).

Histopathological changes in mice skin in Groups I and II (control) showed little differentiation from basal cell layer and in connective tissue, a scarce fibroblast migratory activity was shown as signs of progressive physiological healing (Figures-2a and b). Groups III and IV that received *M. charantia* gel showed healing activity. Group III on 7 days of evolution, scarce fibroblasts, elastic fibers, and collagen were seen in the dermis. In Group IV, there was evidence of dermal papillae formation with the presence of basal cells, presence of fibroblasts in a horizontal arrangement, and a greater amount of collagen to fill the wound (Figures-2c and d).

In the evaluation of the administration of *M. charantia* cream, histopathological changes on day 7 in Group V, in the dermis, there was a considerable presence of fibroblasts and abundant collagen and elastic fibers, the same as in Group VI. In addition, the restitution of the epidermis with papillary projections was observed, indicative of an optimal reparative process attributable to the effect of *M. charantia* cream (Figures-2e and f).

## Discussion

This study presents the wound healing potential of topical gel and cream formulations based on *M. charantia* acetonic extract, applied to induced wounds in mice. Histopathological results in control group shows very few basal cells were observed at the level of epidermis and in dermis, connective tissue was observed with little migration of fibroblasts and collagen. These findings correspond to progressive and physiological scarring.

Dermal lesions involved migration and proliferation of cells such as fibroblasts, endothelial, epithelial cells, deposition of connective tissue, and wound contraction. Collagen not only confers strength and integrity to the tissue matrix but also plays an important role in homeostasis and epithelialization in the phase of healing [25].

Groups that received topical formulations of *M. charantia* show greater healing activity. In Groups III and IV, who received treatment with 1% gel, show continuity of reepithelialization at the level of epidermis with the formation of dermal papillae with the presence of basal cells; and in the dermis, a small number of fibroblasts and a greater amount of collagen were observed. Groups V and VI received treatment with 1% cream, evidence of recovery of epidermal layers: Corneal, granular, spinous, and cell migration to form papillary projections; and in the dermis, there were abundant fibroblasts and presence of collagen, attributable to the effect of cellular regeneration and healing of *M. charantia* cream. These results are comparable with healthy skin in terms of the appearance of collagen and cellularity in the dermis, which evidence epithelialization [26].

Results of this study suggest that the extract accelerates wound healing by inducing fibroblast migration and, in turn increases collagen production in the affected area. In the initial stages of wound healing, fibroblasts play a fundamental role by migrating to the wound area and inducing synthesis of a new extracellular matrix in fibroplasia and granulation tissue formation [27-29]. Important to consider in the proliferation phase is that when collagen has completely filled the wound, the fibroblast activity will stop and this is a physiological process in the human body to prevent excessive wound healing; and under the influence of growth factors and hydrolytic enzymes released by macrophages, fibroblasts proliferate and produce a large amount of collagen [29,30]. The amount of collagen fibers deposited in the tissue can be used as a marker of mechanical resistance of connective and epithelial tissue, which form a support structure to promote wound healing and for maintaining tissue strength in the wound area [31]; also reducing the chances of opportunistic infections in the area. However, excessive formation of collagen or granulation tissue can lead to abnormal scar formation, which is controlled by TGF-β [32].

The previous study has shown that topical application of an ointment with the extract of *M. charantia* fruit improves the expression of TGF-β in a healing model in diabetic rats [33]. The healing activity of *M. charantia* was also reported in the oil obtained from *M. charantia* at a dose of 100 mg/kg and demonstrated an important wound healing activity in buccal mucosa of rats [34].

Based on the results obtained, it is postulated that components present in *M. charantia* acetonic extract, such as flavonoids, steroids, and terpenes, could be responsible for promoting wound healing activity and that is due to the greater effect in cream formulation. It would be mainly due to the fact that those compounds are soluble in oily form of the formulation. Aglycone flavonoids are generally extracted with less polar solvents, such as acetone, and thus, acetone has been shown to be the best of five solvents, including water, for extracting flavonoids from *M. charantia* [35]. Flavonoids have played an important role in wound healing due to their anti-inflammatory potential through inhibition of NFκB synthesis [36].

The use of traditional medicinal plants for wound healing is based on their antiseptic, astringent, anti-inflammatory, and antimicrobial properties [37]. This antimicrobial activity is related to facilitating the wound healing process, since open wounds are particularly prone to infections, especially by bacteria, and they also provide an entry point for systemic infections [25]. The antimicrobial property was reported in the acetonic extract of *M. charantia* leaves as it potently inhibited the growth of *Staphylococcus aureus*; this effect is related to the presence of tetracyclic triterpenoids and their glycosides, most of which are known as cucurbitanes and are known for their bitterness and diverse biological effects [38].

Antioxidant activity reported in *M. charantia* [39,40] is also related to the process of accelerating wound healing, since in the normal physiology of wound healing, it depends on low levels of reactive oxygen species and oxidative stress [41]; therefore, overexposure to oxidative stress leads to poor wound healing [42].

## Conclusion

*M. charantia* gel and cream have been shown to accelerate the wound healing process induced in the skin of mice, being 1% cream formulation the most effective treatment. It is postulated that the healing mechanism of *M. charantia* is related to phytoconstituents such as flavonoids, steroids, and cucurbit triterpenes; they exert antioxidant and antimicrobial effects, which contribute to the optimal healing process.

## Authors’ Contributions

WAS: Collected the plant species, entered it to the herbarium, and produced the first draft. WOS and JLC: Prepared cream and gel formulations. CRS and AAC: Performed organ harvesting for histopathological analysis. VEVT and JH: Performed the statistical analysis and the preparation of images. AAC and CLA: Kept the animals during the investigation and administered treatments. VEVLT and MVG: Carried out the preparation of extract. All authors reviewed, edited, read, and approved the final manuscript.
